# An alternative technique for downsizing a modified Blalock‐Taussig shunt

**DOI:** 10.1002/ccr3.1944

**Published:** 2018-11-28

**Authors:** Dimitrios Bobos, Meletios Kanakis, Evangelia Grisbolaki, Nicholas Giannopoulos

**Affiliations:** ^1^ Department of Paediatric and Congenital Heart Surgery Onassis Cardiac Surgery Centre Athens Greece

**Keywords:** B‐T shunt, conduit, pulmonary overcirculation, transposition of great arteries

## Abstract

An alternative surgical approach for downsizing an existed modified Blalock‐Taussig shunt is described as a reoperation in a hemodynamically unstable patient. This method was selected in order to minimize the surgical manipulations in the setting of a critically ill infant.

## 1 TECHNIQUE

**Figure 1:**
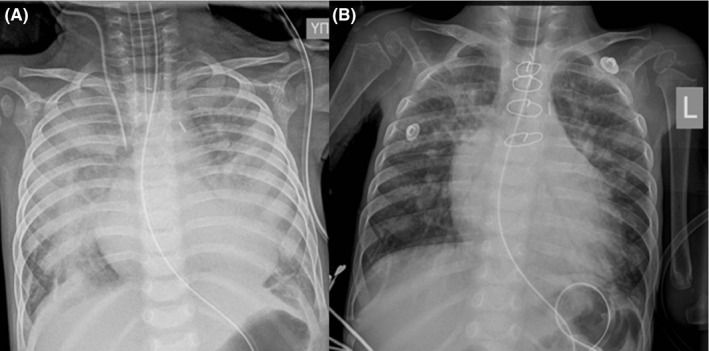
Chest X‐ray showing the pulmonary edema (A) and the improvement on the postoperative day 6 (B)

Modified Blalock‐Taussig (B‐T) shunt has critical impact on the potential outcome of future surgeries on the same patient.^1^ A 10‐month‐old boy, 8.4 kg, with d‐transposition of the great arteries (TGA), VSD, and subpulmonary obstruction, underwent a modified B‐T shunt with a 5‐mm conduit. Postoperatively, the patient developed pulmonary overcirculation due to oversized B‐T shunt (Figure 1A). Critical status of our patient entailed minimal surgical manipulation, so a smaller conduit was tightly wrapped around the previous B‐T shunt off pump.

**Figure 2:**
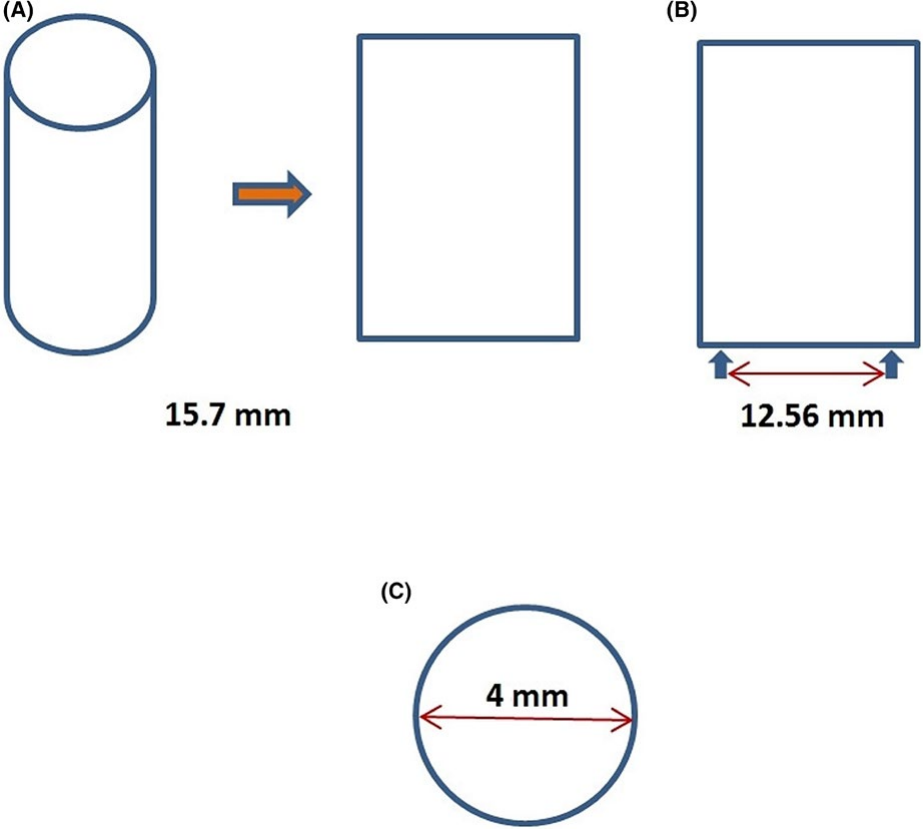
A, Schematic representation depicting a longitudinally opened 5 mm graft in an oblong shape; the width of this corresponds to the perimeter of the graft. B, Schematic representation showing the marked width, which corresponds to the desirable perimeter of a 4 mm graft. C, Schematic representation of the created downsized graft by multiple 6‐0 prolene in an axial level, which shows the created eversion of the pre‐existed graft (perimeter = 2πR = πδ)

A 5‐mm Gore‐Tex was used again. The graft was longitudinally incised creating an oblong shape; the width corresponded to the perimeter of the graft (15.7 mm; Figure 2A).

Perimeter had to be the same as of the 4 mm graft, so we have marked the width of this oblong to the desirable perimeter (12.56 mm; Figure 2B).

**Figure 3:**
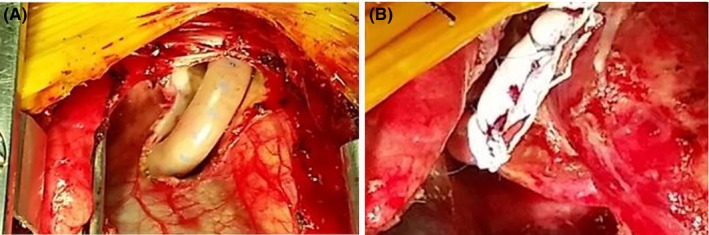
Intraoperative photograph showing the existed B‐T shunt (A) and the created downsized girdling graft (B)

Dissection of the existed shunt from edge to edge was performed (Figure 3A) and then girdling with the new one by suturing it in the marked diameter. Interrupted “U‐shaped” 6‐0 prolene sutures (Figure 3B) were placed creating eversion of the existed graft to prevent thrombosis (Figure 2C).

The patient had an uneventful recovery, and the shunt was functional 6 months later (Figure 1B).

## CONFLICT OF INTEREST

None declared.

## AUTHOR CONTRIBUTION

DB: had the main idea for this alternative surgical approach and helped to draft the manuscript. MK: participated in the main idea for this surgical approach and in the design of the manuscript and drafted the manuscript. EG: participated in the design of the manuscript. NG: conceived of the manuscript idea and participated in its design. All authors read and approved the final manuscript.
